# Artificial neural network for flood susceptibility mapping in Bangladesh

**DOI:** 10.1016/j.heliyon.2023.e16459

**Published:** 2023-05-23

**Authors:** Rhyme Rubayet Rudra, Showmitra Kumar Sarkar

**Affiliations:** Department of Urban and Regional Planning, Khulna University of Engineering & Technology (KUET), Khulna, 9203, Bangladesh

**Keywords:** Flood, Machine learning, Geographic information system, Remote sensing, Bangladesh

## Abstract

The objective of the research is to investigate flood susceptibility in the Sylhet division of Bangladesh. Eight influential factors (i.e., elevation, slope, aspect, curvature, TWI, SPI, roughness, and LULC) were applied as inputs to the model. In this work, 1280 samples were taken at different locations based on flood and non-flood characteristics; of these, 75% of the inventory dataset was used for training and 25% for testing. An artificial neural network was applied to develop a flood susceptibility model, and the results were plotted on a map using ArcGIS. According to the finding, 40.98% (i.e., 499433.50 hectors) of the study area is found within the very high-susceptibility zone, and 37.43% (i.e., 456168.76 hectors) are in the highly susceptible zone. Only 6.52% and 15% of the area were found in low and medium flood susceptibility zones, respectively. The results of model validation show that the overall prediction rate is around 89% and the overall model success rate is around 98%. The study's findings assist policymakers and concerned authorities in making flood risk management decisions in order to mitigate the negative impacts.

## 1 Introduction

An influx of water (or, in rare circumstances, other fluids) that fills typically dry ground is referred to as a flood. Flooding is among the most destructive and expensive natural disasters. Floods are an important concern in agriculture, civil engineering, and public health and are a topic of research in the science of hydrology. It has serious economic and environmental repercussions, including the destruction of farms, the reduction of crop productivity, and regional freshwater shortages [1]. A flood-borne disease affects upwards of 33% of the world's territory, where nearly 82% of the world's residents live [2]. Flooding can cause other related disasters like landslides and different diseases like typhoid, cholera, etc. Both low-lying and high-lying terrain are susceptible to flooding. Water managers can find the most useful variable in flood control by analysing floods and their correlations with explanatory variables [3]. Due to a lack of access to sophisticated flood evaluation techniques for forward planning, flood losses could not be prevented despite having significant financial and developmental effects. There are several ways to describe and assess the risk of flooding [2]. Understanding how the system functions in practise during flooding occurrences is the first step towards reducing the effects of floods. Therefore, with improved management of the river basin, the degree of danger may be lowered even in the most complex circumstances that may occur at the flood peak [4].

Different methods have been put forth to assess the likelihood of a flood disaster depending on the system's vulnerability and hydrology [5]. Some studies have used AHP (Analytical Hierarchy Process) to detect flash floods [2,6]; some research has employed the Fuzzy Analytical Hierarchy Process (FAHP) to predict the development of food insecurity [7]; hydraulic models are carried out in HEC-RAS [4], Mike Urban [8] stimulating floods as well as impact assessment using HEC-RAS and comparison with MODIS images [9]. Research using machine learning techniques has gained popularity in flood prediction and monitoring. In some cases, machine learning approaches such as XGBoost [5,10,11], a tree-based supervised algorithm, have been used, as have KNN (K-Nearest Neighbours) [12,13]. The support vector machine (SVM) is commonly used in flood prediction models [14], and random forest and boosted tree models [15,16] have been used to assess flood susceptibility in recent years. Despite the success of the machine-learning technologies mentioned above as excellent numerical tools for engineering risk assessment, there is still a rising interest in developing a more accurate predictive risk model. An essential fact is sometimes overlooked: the resolution and type of DEM (digital elevation model) data that has been used while extracting points [17]. Studies found that the 30 m resolution of DEM provides higher accuracy than others in machine learning models [18]. Last but not least, one of the machine learning approaches used the most in engineering risk assessment is the artificial neural network (ANN) [19]. An ANN model that integrates GIS (geographic information system) and remote sensing has been shown to be more reliable and precise in terms of prediction [5].

Bangladesh is regarded as one of South Asia's main estuaries. The delta is formed by the confluence of three of the world's most significant river systems, the Ganges, Brahmaputra, and Meghna (GBM), and their tributaries [2]. Bangladesh's unique geographical position makes it vulnerable to a variety of flash flooding occurrences. These include (1) rivers, (2) pluvial, (3) flash, (4) tidal, and (5) storm surge-induced foods [20]. With flooding events, many contagious and waterborne diseases become more prevalent. This catastrophe affects, on average, 5 million people annually [21]. The devastating floods that struck Bangladesh in 1954, 1955, 1974, 1987, 1988, 2004, 2007, 2014, and 2016 resulted in significant property damage and fatalities. An assessment of the flood's damage was made, and it was estimated to have cost 0.85 billion USD (71.55 billion BDT) in financial terms [2]. In Bangladesh, there are around 461 distinct types of wetlands, with Tanguar and Hakaluki haors being the biggest and most well-known [22]. The term “Haor” refers to circular or elliptical-shaped depressed marshes that are mostly found in the districts of Sunamganj, Kishorganj, Sylhet, Netrokona, and Brahmanbaria [23]. These wetlands are essential to the country's economy, environment, and way of life. Wetlands are crucial for life and livelihood, and many Bangladeshis depend on these natural resources for a living. Flash food occurs during the pre-rainy season months of March, April, and May in the north-eastern region of Bangladesh, recognized as the Haor (wetland) region. The confluence of Himalayan glacier melt water and significant rainfall in the neighboring undulating plains of northeastern India known as the Haor (wetland) area is thought to be the source [24]. According to the Department of Disaster Management (DDM) report, the 2017 flash food crisis affected 850,088 families (29.3%) in seven Haor districts [2]. The district with the smallest proportion of infected homes, 18.9%, was Habiganj, followed by Shunamganj (39.2%), Sylhet (35.7%), and Netrokona (34.9%) [25]. About 800,000 metric tonnes of Boro rice, valued at 450 million dollars, were placed in food storage to compensate for the loss of food grains due to floods [2]. Flood susceptibility mapping and assessment are critical components of flood mitigation and preventative approaches because they identify the most vulnerable regions based on physical elements that influence the chance of flooding. Building a flood susceptibility model for this region can play a vital role in mitigating flood loss, improving land use management, and making the local people more concerned.

Various studies have been done in this area regarding flooding. Several studies like change in agricultural land at Tanguar Haor due to flash floods [22], level of vulnerability and resilience in the northwestern Haor region by social survey [23], flood characterization using flood index [24], and analytic hierarchy process (AHP) in conjunction with GIS techniques to identify flood hazard zones [2], evaluation of flood-exposure risk adjacent to Bangladesh's river network using multitemporal satellite imagery [26], artificial neural network (ANN), logistic regression (LR), frequency ratio (FR), and analytical hierarchy process (AHP) for flood assessment [27], hybrid machine learning algorithms in delineating multi-type flooding [28], flooding and its relationship with land cover change, population growth, and road density [29] have been done in this region over the years. But none of these studies have used machine learning algorithms for flood susceptibility modelling at the regional level in Bangladesh. Machine learning approaches may improve regional flood risk mapping. Machine learning models can better represent flood susceptibility in regional studies with many environmental and socio-economic factors by incorporating complex relationships between variables and integrating multiple data sources at the regional level. In this study, artificial neural networks (ANN) and GIS will be used for the creation of a flood susceptibility model of the Shylet division, which includes several haors, bills, and rivers. Specific objectives of the study are: i) Flood susceptibility mapping using ANN and GIS ii) accuracy assessment using sensitivity analysis and the ROC curve; iii) using pairwise plots and generalized plots to see the relationship between different factors and influences causing flooding. This research would be able to identify local changes in flood susceptibility that had not previously been detected in lower-resolution investigations by employing high-resolution topographic data. More precise findings may be obtained from flood susceptibility mapping if the spatial resolution is increased for a smaller area. This is the first study to detect flood susceptibility at the regional level of Bangladesh using ANN and to show relationships between factors and their influence using pairwise and generalized plots. This study can contribute to the existing body of knowledge by providing a more detailed and localised understanding of flood susceptibility for disaster management and decision-making in a critical region like the Sylhet division of Bangladesh with an advanced machine learning algorithm.

## 2 Materials and methods

### 2.1 Description of the study area

**Fig. 1:**
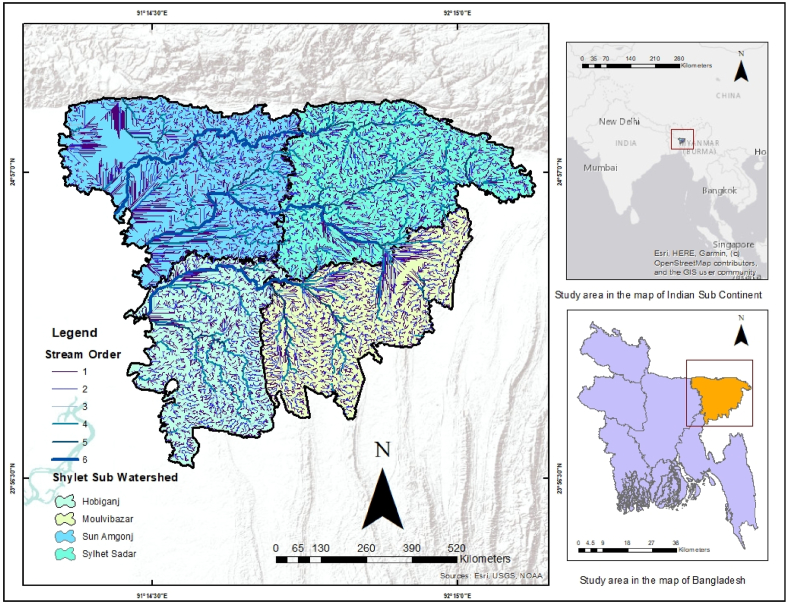
Study area.

Sylhet division is situated between 23°58′ W latitude and 25°12′ W latitude and 90°56′ E to 92°30′ E longitude (Fig. 1). It is bordered by Meghalaya state of India to the north, Tripura state of India and Brahmanbaria district of Bangladesh to the south, Assam and Tripura states of India to the east, and Netrakona and Kishoreganj districts to the west. The division of Sylhet has a total area of 12,558 square kilometres. The size of the district of Sylhet is 3452 square kilometres; the district of Sunamganj is 3670 square kilometres; the district of Habiganj is 2637 square kilometres; and the district of Moulvibazar is 2780 square kilometres. Sunamganj is the largest district in this division in terms of land area, whereas Habiganj is the smallest [30]. According to the 2022 population census of Bangladesh, the total population of the Shylet Division is around 16 million. It is made up of a patchwork of wetland habitats such as rivers, streams, and irrigation canals, as well as vast expanses of seasonally flooded farmed plains and countless haors and beels. This region is made up of about 400 haors and beels that vary in size from a few hectares to many thousand hectares [31]. These haors and beels support significant shelter, food, and commercial fisheries, while the lake margins are periodically flooded, allowing for significant rice cultivation. Along with rivers, canals, and the floodplain, harbours and bays are key sources of fish production. However, due to siltation and excessive fish harvesting to meet the demands of expanding populations, fish productivity from this source is gradually declining [32].

### 2.2 Description of the data

Different methods are used when choosing the variables to measure the susceptibility to flooding. For example, researchers like [2] have used several factors like NDVI, distance from a river, soil type, TWI, rainfall, LULC, etc. On the contrary [33], have considered slope, land use, elevation, drainage density, soil type, and rainfall as food hazard factors. For ML approaches, factors like elevation, slope angle, slope aspect, flow accumulation, flow direction, TWI, rainfall, and NDVI have been used [5]. This study identifies eight criteria based on a review of previous studies conducted in an indistinguishable environment. The majority of the data and statistics used in this study came from secondary sources, while the historical food points were acquired via a survey carried out by the Bangladesh Water Board, as reported in Table 1. Landsat-9 pictures are used for image processing, and a digital elevation model (DEM) is created for the study area.

**Table 1:** Data source.

Category	Source	Resolution	Data Type
DEM (digital elevation model)	NASA Earth Data [34]	30 × 30 m	Raster
Slope	Extracted From DEM	30 × 30 m	Raster
Aspect	Extracted From DEM	30 × 30 m	Raster
Curvature	Extracted From DEM	30 × 30 m	Raster
Roughness	Extracted From DEM	30 × 30 m	Raster
SPI	Extracted From DEM	30 × 30 m	Raster
TWI	Extracted From DEM	30 × 30 m	Raster
LULC	USGS [35]	10 × 10 m	
Historical Flood Points	Bangladesh Water Development Board		Vector

### 2.3 Training and testing points

**Fig. 2:**
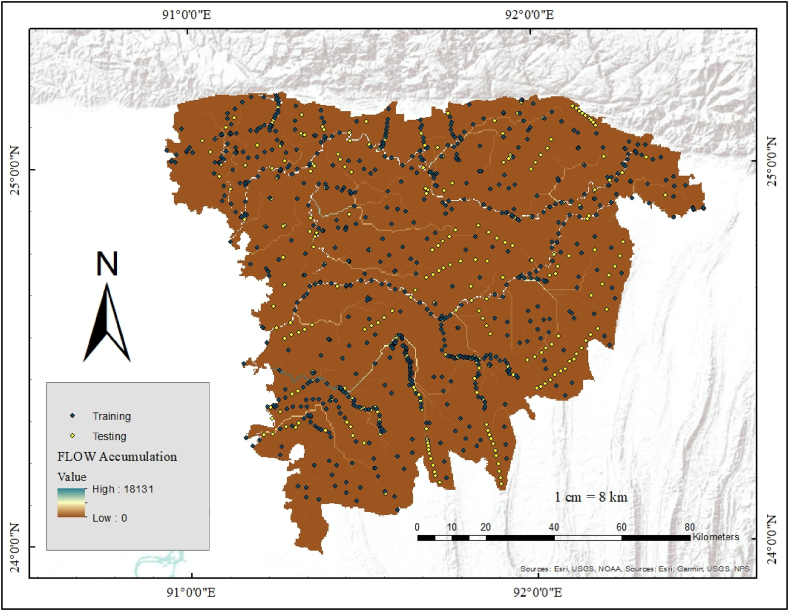
Training and testing points.

Flood prediction mapping relies on the identification of flood-controlling factors for precision. In this work, around 1280 samples were taken at different locations. From these locations, some areas faced flooding, and some areas did not face flooding in previous years. Flood points were taken from and BWDB and non-flood points were generated from buffer application using a safe distance following the work by Ref. [36]. The obtained data were then processed and categorized into training and testing sites using GIS software and a randomization technique (Fig. 2). 75% of the inventory dataset was used for training and 25% for testing. In spite of this, it is determined that a binary classification known as susceptibility mapping divides the food inventory into two categories, such as food points and non-food points. Then, binary digits such as 0 and 1 are utilized to identify flood cases. The binary number 0 was used to indicate that the site was flooded, whereas the binary number 1 was used to indicate that the site was not flooded. Around 1000 points were used as training data, and around 280 points were used as testing data. Historical flood points were also used to identify the areas that were not flooded using GIS model builder operations.

### 2.4 Description of parameters

**Fig. 3:**
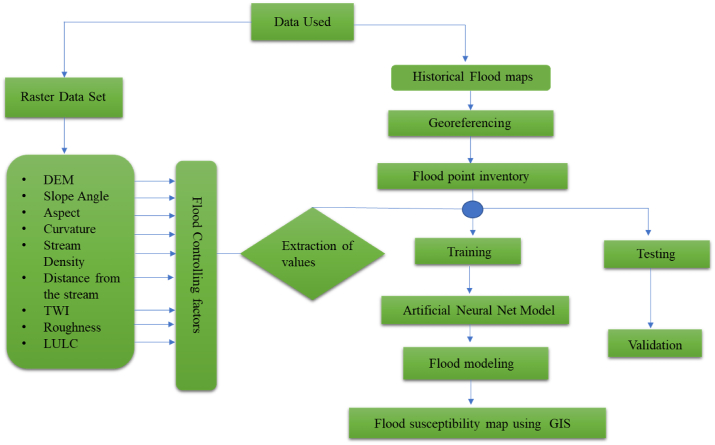
Methodological flow chart.

Several software packages, including ArcGIS and R, were used in this paper to identify flood-prone areas. Different flood parameters like slope, TWI (topographic wetness index), SPI (stream power index), aspect, curvature, roughness, elevation, and LULC (land use land cover) were used in this study. The accessible datasets were gathered and united into standardized formats and recorded in GIS and ArcMap with the same extension for later processing. The datasets were heterogeneous in both kind and extension (Fig. 3). Training and testing points were used to extract the value of each point. An extract values by point tool was used to extract each value. How the values are extracted from the raster is determined by the interpolation setting. Extraction of the precise cell value from the input locations is the default choice. All the values are then saved as an Excel data sheet. The initial datasets were obtained, and R was used to process them (scale, normalize, and model predictions). For the training and validation datasets, thematic layers of 0 and 1 were constructed and converted into “no” or “yes” map layers. The flood susceptibility map was modelled using training data, and the model was validated using testing data. The processing of each geographic layer, as well as the principles and methods underlying the machine learning algorithms employed in the current work, are discussed below.

#### 2.4.1 Elevation

Elevation is vital in managing the direction and route of the overflow flow, as well as the depth of the water level [33]. The elevation map depicts altitude ranges ranging from 0 to 783 m. Elevation is another often utilized metric and one of the primary elements influencing an area's floods [5]. Flood disasters compared to landslides, that are more likely to happen at higher altitudes, are more likely to happen in low-altitude areas [37]. In general, flooding and waterlogging are more likely at lower elevations because runoff from higher terrain collects there. The elevation raster map was made with ArcGIS program using (DEM) data. The elevation raster was divided into four subsets.

#### 2.4.2 Slope angle

The degree of inclination and sharpness with regard to the horizontal plane determine the slope [38]. The slope of an area and the velocity of flowing water may be related in a positive way. A steeper slope in a region may speed up precipitation-related runoff. As the gradient increases, surface runoff increases rapidly, while infiltration decreases. As a result, places with an abrupt fall in slope have a greater likelihood of flooding because a vast volume of water gets immobile, resulting in a serious flood disaster situation [5]. The slope factor was calculated using data from the (DEM). The slope raster was created in ArcGIS using the slope tool. Then slope values of testing and training points were extracted and inputted in the excel sheet.

#### 2.4.3 Aspect

This feature, also known as slope orientation, detects the downslope direction [39]. The slope aspect represents the surface's steepness and is divided into three categories based on color intensity or saturation. In the output aspect-slope raster, the pixel values indicate a mix of aspect and slope. This component is an important consideration when creating flood susceptibility maps [40]. The aspect values were extracted through training and testing points.

#### 2.4.4 Curvature

In the majority of literary works, curvature is recognized as one of the flood-conditioning variables [41]. Curvature values, which are the rate of change in slope gradient in a specific direction, demonstrate how the topography is formed [15]. A positive curvature suggests an upwards convex gradient, a zero value shows no curvature, and a negative curvature indicates an upwardly concave gradient [42].

#### 2.4.5 TWI (topographic wetness index)

The TWI is a physical manifestation of flood inundation zones, which are an important part of a river watershed. A catchment's TWI indicates two kinds of measurements: flat lands and hydrographic places. The TWI is commonly used to quantify topographic influences of hydrological processes [5]. In determining foods, the topographic wetness index (TWI) calculating process is less expensive than typical hydrodynamic models [43]. TWI is calculated by analyzing DEM data. After extracting the slope and flow accumulation raster, the following Equation (1) is calculated using a raster calculator [44]:

(1) TWI = ln(α /tanβ+c)

In which the total upslope contribution area draining via a point (per unit contour length), and tan is the slope angle of the place. It influences the geogsraphical distribution of soil moisture, and groundwater flow frequently follows the contour of the land. TWI is considered a significant factor in this study. High wetness index locations occur where there is a combination of low slope and increased flow accumulation and, as a result, may indicate flood-prone places [45].

#### 2.4.6 SPI (stream power index)

The erosive power of streams is reflected in the stream power index (SPI). The following syntax was used to calculate the SPI in the GIS environment's “Raster Calculator” [46]:

(2) SPI = Ln (Flow Accumulation) × (Slope)

A high positive SPI suggests that the erosive index is significant., whereas a low positive or negative SPI indicates a low erosive index or a high deposition index [46]. In this study, Flow Accumulation was calculated from DEM. Then raster calculation tool was used to calculate the following Equation (2). Ln of Flow Accumulation was multiplied with slope.

#### 2.4.7 Topographic roughness index (TRI)

One of the key determinants of flood occurrences is the topographic roughness index (TRI). The roughness index measures the altitude between neighboring cells in a digital elevation model (DEM). Roughness index frequently used to describe topographic undulations. The rougher the surface, the greater the undulations, and vice versa. Long-term weathering and erosion processes continually transform a mountainous region's rocky terrain into a smooth and flat surface, resulting in a region's undulating topography [47]. It relies on the study basin's local topography. The lower the TRI number, the higher the likelihood that a flood will occur [48]. Roughness is calculated by using raster calculator tool in GIS. First of all, focal statistics minimum, maximum and mean were calculated using focal statistics tool. Then the following Equation (3) was applied in the raster calculator [47]:

(3) TRI= FS_(mean)_ - FS_(min)_ /FS_(max)_ - FS_(min)_

#### 2.4.8 LULC

Land use and land cover (LULC) are important contributors to surface runoff and possible flooding in a watershed. Different forms of land cover have distinct effects on the occurrence of food risks [49]. The capability of the soil to absorb and moisture retention is greatly influenced by land cover, such as agricultural land and vegetation. Human activities including pasture, deforestation, urbanization, agriculture, and landfills increase the frequency and intensity of flash floods. Agricultural land and vegetation can better absorb runoff rainfall than bare soil. The presence of lush vegetation slows the transfer of water from the surface to bodies of water, minimizing runoff. Concrete and other solid substances, on the other hand, absorb the least quantity of water. Roads, constructions, buildings, and settlement areas limit the soil's ability to absorb water and increase runoff. This highlights the importance of land usage land cover in assessing the likelihood of food supply [2]. This study demonstrates how important land use and cover are in determining the likelihood of flooding. Landsat 9 satellite images were used to create the land use and cover map. Input of raw data into GIS comes first. In order to combine the bands, composite bands and mosaic band tools were used. The unsupervised image classification approach is then used to identify the various types of land cover. Following the identification of 25 classed categories, five distinct classes—including waterbodies, agricultural land, vegetation, built-up areas, and vacant land—were observed.

### 2.5 Method of flood susceptibility mapping

The artificial neural network (ANN) is regarded as a quantitative black-box method that aims to imitate the working biological nervous system of humans. Forecasting and environment nonlinearity analysis may be researched utilizing this efficient and cost-effective machine learning strategy. The GANN (GIS and ANN) model is still infrequently utilized for flood susceptibility assessment, despite the fact that in earlier studies the ANN has also been successfully used for flood analysis [5,50] the GANN (GIS and ANN) model is still is not frequently used for flood susceptibility assessment. Because of its simplicity in terms of data collection requirements, the ANN has been suggested as a replacement to physically-based models [51]. Using the digital elevation model and LANDSAT 9 data, many parameters, such as elevation, slope angle, aspect, SPI, curvature, Roughness TWI, and LULC, were created. For the purpose of comparing flood vulnerability, historical flood data were used. The R Project software is compatible with nnet and neuralnet packages for feed forward ANN [52]. Neural net package was used in this study to get the expected outcome. Using this package, it is possible to build several hidden layers with any number of neurons; additionally, the user can select from a small number of accessible learning methods and activation functions [53]. The biological neuron and its reduced properties are the fundamental unit of artificial neural networks. These were developed as a simple mathematical model replicating human brain function [54]. It is composed of n inputs, resulting in the vector x= (x1, …. xn). Every input is multiplied by the weight parameter, which can be either positive or negative. Weight x0, which represents the bias, rates another input neuron x0 = 1. The total of all weighted inputs yin represents the internal potential of the neuron [53]:

(4) $$Yin=\sum_{i=0}^{n}WiXi$$

The following Equation (4) is used to calculate the potential of the neuron. To produce the neuron's final output, the weighted sum is passed via a neuron activation function y = f (y in). This, in turn, can act as a stimulus for neurons in the neural network layer below. A neural network is formed when neurons are linked together. The linking mechanism is set up in such a way that the output of one neuron becomes the input of another. The neurons in the network are organized into layers [55]. Each network has an input layer, an output layer, and any number of hidden layers. The ability to modify the weights between neurons is a key property of neural networks. The weights in the network are reinforced or weakened based on the correct or incorrect answers [53]. Learning algorithms are classified into three types: supervised, unsupervised, and reinforcement. A network model is a multilayer neural network in which each neuron is represented as a training algorithm. Neuron activation functions in multilayer perceptron are differentiable continuous functions, with the sigmoid function being the most widely used [56].

(5) $$f\left(x\right)=\frac{1}{1+e^{x}}$$

Equation (5) is used to calculate the sigmoid function. Multilayer perceptron result in complete neuron connectivity - each neuron in the layer is connected to all neurons in the preceding (following) layer. Using the digital elevation model and LANDSAT 9 data, many parameters, such as elevation, slope angle, aspect, SPI, curvature, roughness TWI, and LULC, were created and used as an input in neural network package. Aspect and LULC data were transformed from categorized data to numerical data several neurons. Aspect data was categorized from a to h with an interval of 45. In the case of LULC data each class such as agricultural land, vegetation, waterbody, vacant land and buildup area were categorized from l1 to l5. While selecting non-flood points, we have used buffer application using safe distance but real-life scenario can be different. In the case of model selection there are more powerful deep learning models which provides more accuracy.

## 3 Results

### 3.1 Flood susceptibility factor

**Fig. 4:**
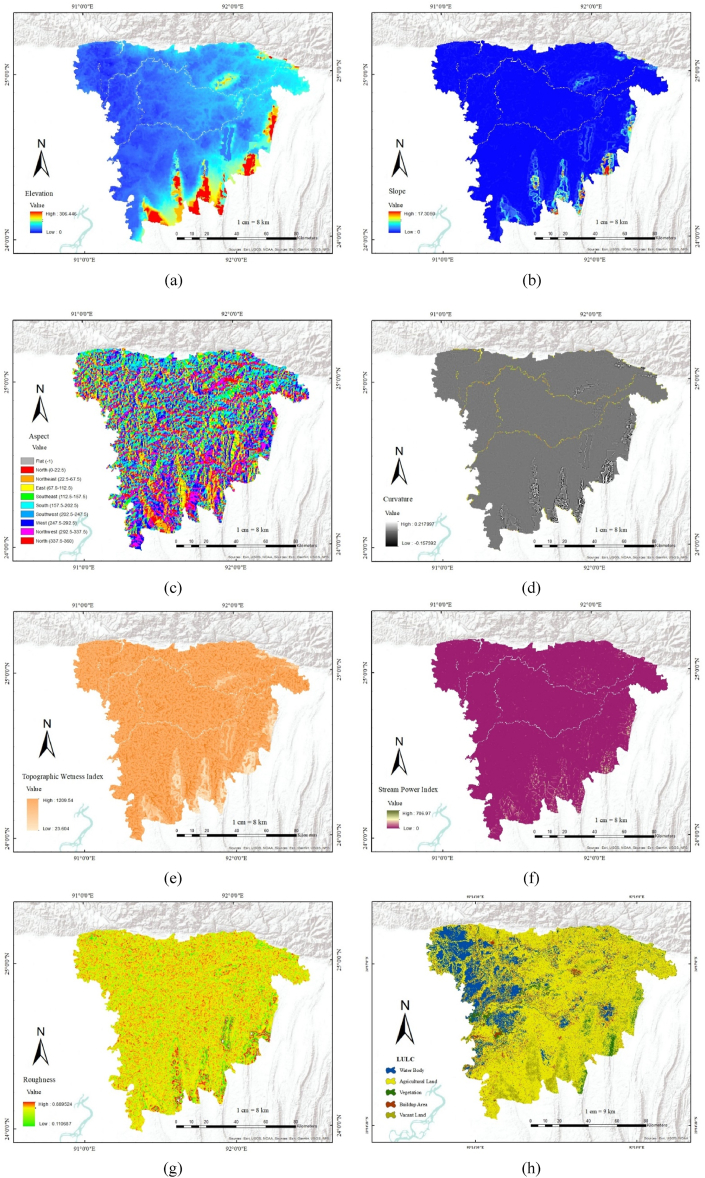
(a) Elevation; (b) slope; (c) aspect; (d) curvature; (e) TWI; (f) SPI; (g) Roughness; (h) LULC.

The digital elevation model is critical for identifying food-prone areas. The red section has the highest elevation value, while the blue portion has the lowest elevation value. The southern portion of Shylet Division holds the greater elevation value because that is where the majority of the steep terrain is located. Most of our study area is low ground with a variety of water bodies, so the elevation values are generally low. The highest value of our study area is 306 and the lowest value is 0 (Fig. 4 (a)). Slope is an important factor in detecting floodplain areas. The results show that the eastern part of the Shylet division has a higher slope angle value, while the northern side is mostly flat (Fig. 4 (b)). It is an important fact to notice as the rainwater moves from hilly areas to flat areas. The highest value of the slope angle is 17, and the lowest value is 0. (Fig. 4 (a)) demonstrates that the preponderance of the research area has elevations less than 15 m, with the exception of a small section in the north-eastern corner. The aspect value is heterogeneous throughout the study area. It ranges from −1 to 360 (Fig. 4 (c)). (Fig. 4 (d)) depicts the curvature value of the area under consideration. The value of curvature ranges from −0.1597 to 0.217997. The north-eastern portion of the study area has a greater curvature value. Furthermore, the majority of the study area has a positive curvature value. In the study area, TWI values vary from 1200 to 23 (Fig. 4 (e)). As the value rises, so does the topographic moisture and the likelihood of flooding. However, as the value decreases, so does the topographic wetness and the likelihood of flooding. The north-eastern part of the region has a cooperative SPI value (Fig. 4 (f)). It is between 706.97 and 0. The roughness ranges from 0.859 to 0.1106. The roughness value varies across the region. The roughness value is low in the northern portion and high in the central and eastern parts (Fig. 4 (g)). (Fig. 4 (h)) shows the LULC map of the study region. As our area contains a lot of wet lands, the percentage is comparatively higher in this region than in other traditional regions. The proportion of the water body changes with the seasons. Low plains are flooded during the rainy season, creating temporary water basins (haor), while the water evaporates over the winter, leaving bare lands [2]. 10% of the total area is buildup area. Agricultural land is the most abundant in our study area, and vacant land is around 20%, where vegetation is around 15%. Water bodies and agricultural land vary throughout the season.

### 3.2 Artificial neural network model outputs

**Fig. 5:**
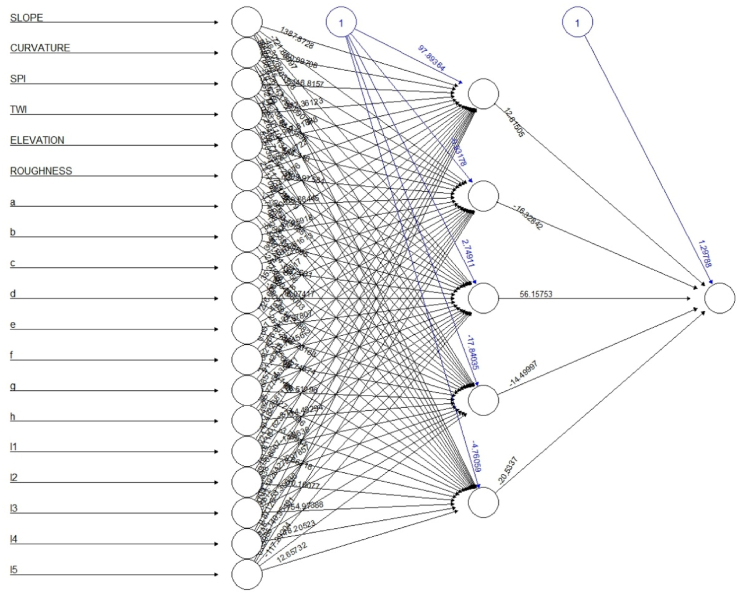
Model structure for flood susceptibility mapping.

**Fig. 6:**
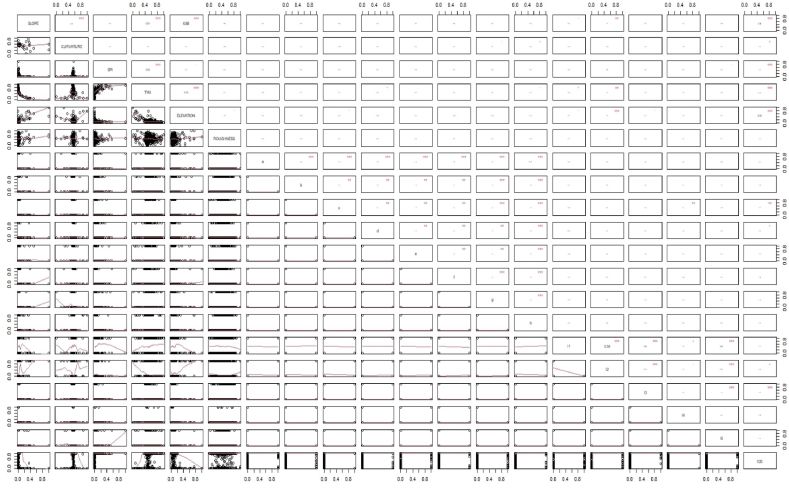
Pairwise plot of training data.

The network design was developed to ascertain the flood susceptibility of the Shylet division; a flood history output layer was required for the model's architecture. This was used to evaluate the model using the mean squared normalized loss performance function, which assesses the network's performance index. Fig. 5 shows three types of layers, such as the input layer, the hidden layer, and the output layer. As the input layer, many different contributing elements are used. Only training data was utilized to create the model's framework. These forms of data were transformed into numerical form since categorical data are challenging for machine learning models to understand. This made the data easier to read for neural network models. The influence of aspect and LULC data for the flood susceptibility model was provided in distinct figures to help readers better grasp the outcome. Fig. 6 shows pairwise plots of factors. A pair plot displays the distribution of single variables as well as the relationships between two variables. Generally, pairwise denotes “occurring in pairs” or “two at once.” Pairwise can also mean “pairwise disjoint.” Random variables with pairwise independence Pairwise comparison is the practice of comparing two items in order to determine which is more advantageous. The X axis of each graph displays the prediction influence from 0 to 1, while the Y axis displays the value. In certain graphs, stars are used to represent the degree of influence. The higher the number of stars, the higher the influence. Example: Slope has a greater level of influence on curvature, as the majority of the forecast value is centered between 0.4 and 0.8, and three stars indicate the level of influence. This approach can also reveal the relationship between slope and other parameters. The third column demonstrates that slope and curvature have no effect on SPI. The last row depicts the influence of factors on the final susceptibility model using a graph, and the column depicts the influence of these factors using stars. Slope, SPI, TWI, elevation, and L3 (a water body) have a greater impact. Slope has a prediction value concentrated between 0.0 and 0.4; SPI has a prediction value concentrated between 0.0 and 0.5; TWI has a prediction value concentrated between 0.2 and 1, with the majority of them concentrated in 0.5; and Elevation has a prediction value concentrated between 0.2 and 1, with the majority of them concentrated in 0.2.

**Fig. 7:**
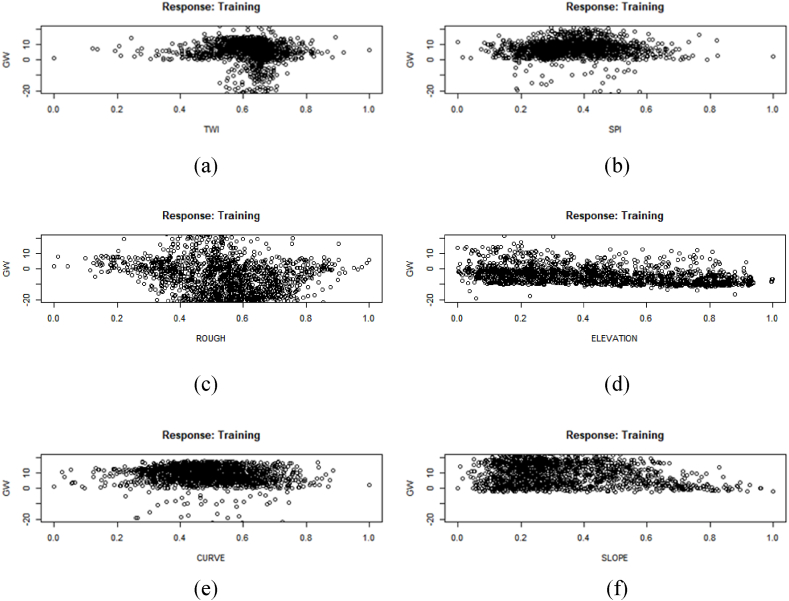
Generalized weight of training data (a) TWI, (b) SPI, (c) Roughness, (d) Elevation, (e) curvature, (f) Slope.

**Fig. 8:**
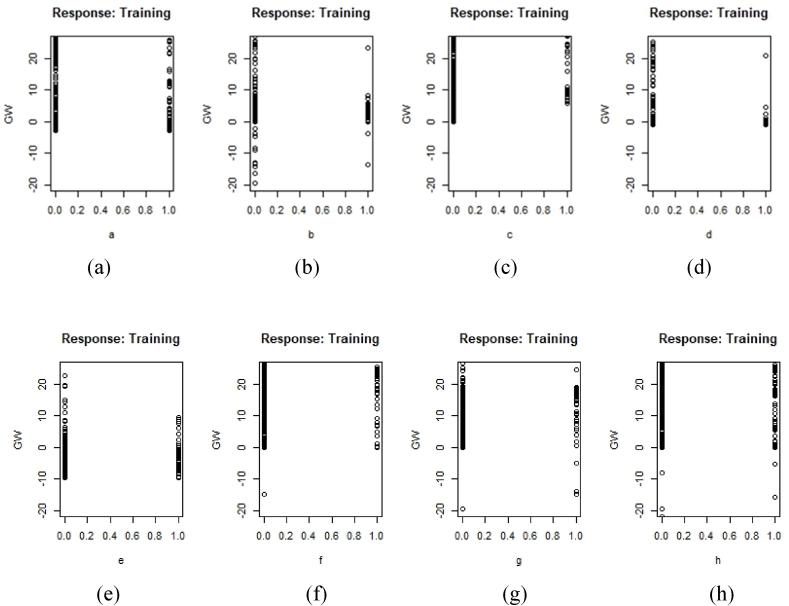
Generalized plot of converted Aspect training data (a) a, (b) b, (c) c, (d) d, (e) e, (f) f, (g) g, (h) h.

**Fig. 9:**
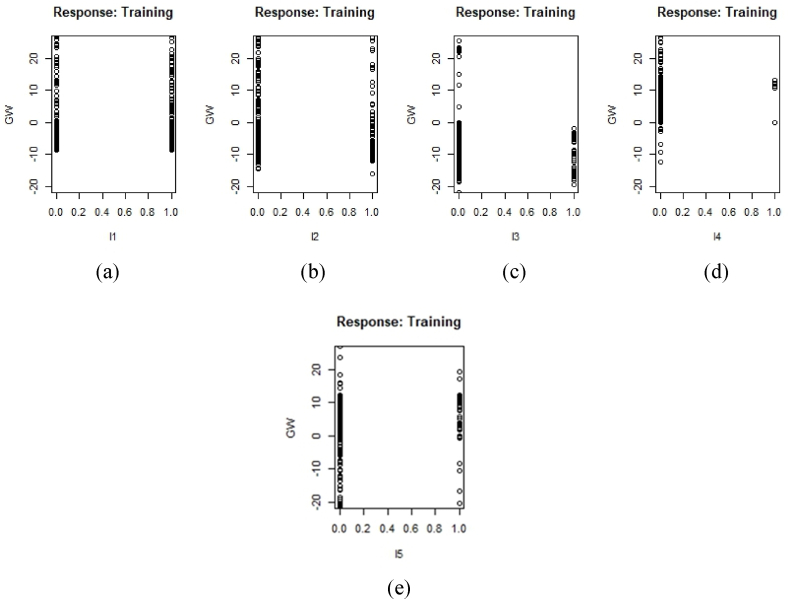
Generalized plot of Converted LULC data (a) l1, (b) l2, (c) l3, (d) l4, (e) l5.

Three stars in the last column indicate a higher level of influence on the prediction model. Generalized weights make it easier to understand each input variable's independent effect on the NN model. A covariate's independent influence is non-linear if the generalized weights for that covariate have a high variance. A covariate is thought to have little impact on the outcome if its generalized weights are close to zero [57]. Fig. 7 illustrates the generalized weight of each element. If the generalized plots are concentrated across the entire graph, the graph will have a greater weight value. As the majority of values fall between −20 and + 20, factors such as slope, SPI, and TWI are given a greater weighting. Elevation carries a heavier weight due to its concentration of positive values between 0 and 20. Fig. 8 shows a generalized plot of converted numerical aspect data. As the majority of data are focused between 0 and 10, it can be seen that aspect data b and d have a lower amount of influence than other data. The remainder are of greater value. The majority of the aspect weight has a positive generalized weight. Fig. 9 shows a generalized plot of converted LULC data. It can be claimed that l4, which is vacant land, has the lowest level of influence because the majority of its values cluster around 0. The remainder of the LULC class has a more generalized plot. Agricultural land (l1) has a positive generalized weight, whereas waterbodies (l3) have a negative generalized weight.

### 3.3 Flood susceptibility mapping

**Fig. 10:**
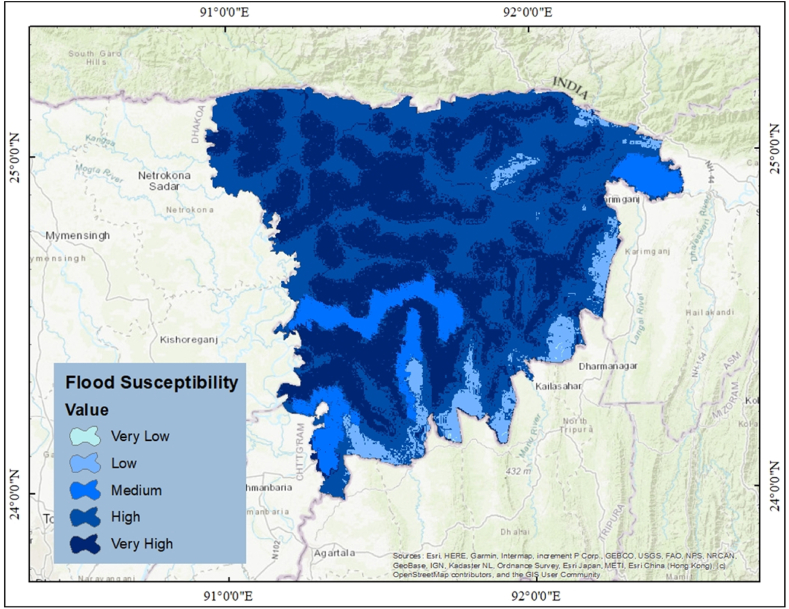
Classification of flood susceptible area.

In addition to being helpful when responding to floods, the flood susceptibility map is valuable for controlling disasters. Since it identifies the most vulnerable locations and offers people adequate time to react to floods in an anticipatory rather than reactive manner, flood susceptibility mapping could be the first step towards flooding mitigation [58]. The total area was classified into five groups, such as very low, low, medium, high, and very high. Fig. 10 depicts the overall susceptibility of the study area. 43.43% of the total area comes under the very high-susceptibility zone, which is around 529,290 hectors; 49.98% of the total area comes under the highly susceptible zone, which is around 609,165 hectors. It is the highest among the group. 6.52% of the total area comes under the low-susceptible zone, which is around 79,497 hectors. Only 0.035% and 0.029% of the area fall into the very low and medium susceptible zones, respectively (Table 2). Blue points depict the historical flood points of the region. It can be seen that areas close to these points have a higher level of flood susceptibility (Fig. 10).

**Table 2:** Classification of Flood susceptible area.

Class	Area (Hectors)	Percentage
Very Low	423	0.03
Low	76170.31	6.52
Medium	182,808	15.00
High	499433.50	40.98
Very High	456168.76	37.43
Total	1218725	100

### 3.4 Model validation

**Fig. 11:**
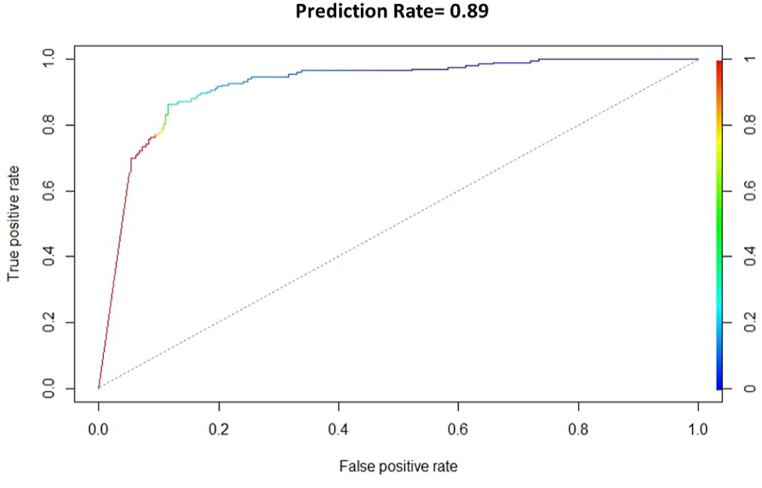
Prediction rate curve.

**Fig. 12:**
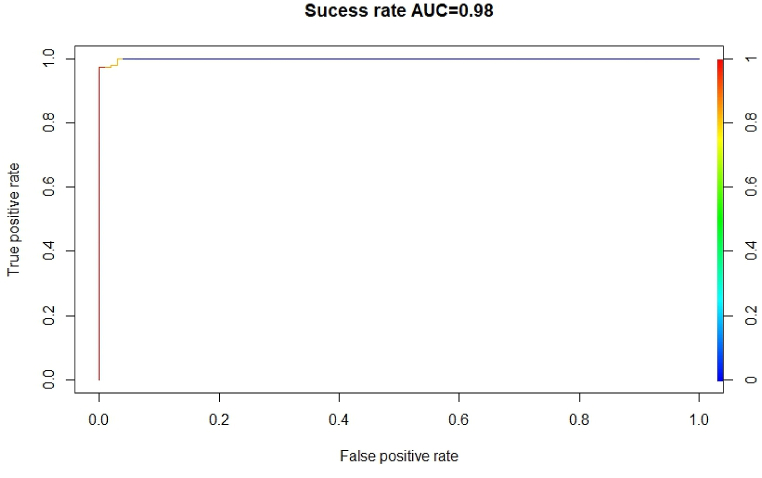
Success rate curve.

To evaluate how well prediction models, operate, they must be checked or validated, and for model validation in this work, we used the receiver operating characteristic (ROC) curve technique for model validation (area under the curve, AUC). This is a critical feature of probability-based mapping and is used in the majority of statistical or probabilistic models. This is a critical feature of probability-based mapping and is used in the majority of statistical or probabilistic models [59]. ROC is the most commonly used and favored kind of model validation, having been utilized in numerous studies, including [[59], [60], [61]]. However, in the current study, all of the models produced prediction maps with AUC values more than 70%, showing trustworthy efficiency. The ROC curve was generated using 25% of the testing data. The results showed that the models used in this study adequately represented the favorable correlations between prediction maps and flood inventory points. In this study, two curves are used to validate the model. One curve is to show the prediction rate (Fig. 11), and another curve shows the prediction rate (Fig. 12). The graph's X axis represents the true positive rate, and the curve's Y axis represents the success rate (Fig. 11, Fig. 12). The results show that the overall prediction rate is around 92% and the overall model success rate is around 98%.

## 4 Discussion

Beginning on the 16th and 17th of June 2022, high upstream precipitation from the Indian states of Meghalaya and Assam has forced rivers in Sylhet to overflow their banks and flood the division. Simultaneously, around 7.2 million people in Sylhet, Sunamganj, and the neighboring northeastern districts were impacted by flood waters, making both the present and the future worrisome. 500,000 people have been relocated to 1432 emergency shelters, while millions more are still homeless in flooded areas [62]. This is one of the more recent events, but these kinds of disasters are pretty common in this region. A model of flood susceptibility can be a very important tool for mitigating flooding in this location.

A susceptibility model was created in this study using the machine learning algorithm ANN. Pair-wise plotting (Fig. 6) shows that factors like slope, elevation, SPI, TWI, and waterbodies have a high level of influence on the prediction of the map, which is pretty common in other machine learning approaches as well [59]. Fig. 7, Fig. 8, Fig. 9 also show generalized weights. The application of generalized weights facilitates the interpretation of the NN model in terms of the independent influence of each input variable. A substantial variance of generalized weights for a covariate suggests that its independent influence is nonlinear. If the generalized weights of a covariate are close to zero, it is assumed that the covariate has little effect on the outcome [57]. Factors like roughness, curvature, and vacant land have low levels of generalized weights as most of the values are concentrated close to zero, while slope, elevation, SPI, TWI, agricultural land, buildup area, vegetation, waterbodies, and most of the other aspects have high levels of generalized weight. Results show that the northwestern parts are the most susceptible to flooding. 40.98% of the total area comes under the highly susceptible zone, and 37.43% of the total area comes under the very highly susceptible zone, which are situated mostly in the northwestern part of the region. The main reason can be described as that most of the high-level terrains are situated in the southern part of the region, and the northern part of the region contains different types of waterbodies. During the time of precipitation, water goes through the high terrain to the low terrain and exceeds the capacity of the waterbodies, causing a flood. This is one of the common reasons for flooding in other studies as well [5,7]. According to our findings, the northwestern districts of Sunamganj and Hobiganj are especially prone to flooding. This is because there are numerous little and large bodies of water that collect rainfall during the rainy season and overflow into the nearby areas. 15% of the total area comes under medium susceptible zone. Only 6.52% of the total area is considered the low-lying area, and most of it is situated in the eastern and southern regions. Because the majority of the eastern section is made up of steep slopes and higher ground, flooding is moderate in comparison to a highly susceptible region. The southern section has the least amount of flooded land compared to the rest of the research area. Previous research conducted throughout Bangladesh using the LR (logistic regression) model, Artificial Neural Network (ANN), and Frequency Ratio (FR) categorized the Sylhet region as a predominantly high, medium, and very high flood susceptible zone (Rahman et al., 2019). In contrast, the ratio of very high susceptible zone to highly susceptible zone is greater in the LR model than in the FR and ANN models. On the other hand, the AHP (Analytical Hierarchy Process) method used by (Haque et al., 2021) for only the stylet region indicates that moderate and high susceptible zones dominate the region, whereas the same model used by (Rahman et al., 2019) for the entire country of Bangladesh indicates that high susceptible zones dominate the Shylet region and the proportion of very high susceptible zones is low. This research closely resembles the research conducted by (Rahman et al., 2019) using LR, FR, and ANN models, but it displays more classes of variation because there is no area classified as low and very low. This is possible due to the varying size of the study area and differences of the used factors. However, an AHP analysis reveals a significant magnitude of difference [27,44]. It can be said that the historical flood has a connection to vulnerable areas. Places where historical floods are concentrated consist of highly susceptible zones, which have not been shown in previous studies done in this region [2]. To validate the result, a ROC curve has been used. A ROC curve has been made by using 25% of the testing data set. It can be seen that the overall prediction rate is 89% and the success rate is 98%, which shows same type in some extend better accuracy than others [27,28,44,48,59].

Numerous studies in different regions of the world have been conducted utilizing diverse decision-making techniques, approaches, and concepts. In this area, the use of machine learning algorithms and GIS in flood susceptibility modeling is relatively new. Machine learning could speed up the processing and interpretation of data, making it a useful tool for applications involving predictive analytics and making maps using GIS more attractive to viewers. Previous investigations indicated that the ML approach used in this study could have a good application in this area [2]. The absence of a physical study and the reliance on secondary sources of data for the majority of the study's analysis are the most significant limitations. Training neural networks necessitates vast amounts of data. To circumvent this limitation, more training datasets have been utilized. Spatial resolution may also be a limitation. The resolution of the input data may affect the accuracy of your results, particularly if there are significant variations in the flood susceptibility at the local level. To surmount this limitation, the sensitivity test by ROC curve is used.

## 5 Conclusion

Machine learning methodologies teach academics and data scientists how to manage large datasets with constrained capacity. In our study, a flood susceptibility map was created using an artificial neural network. The current flood danger in each administrative area might be ascertained using the maps of flood susceptibility. Rapid information gathering about likely target areas could provide information regarding evacuation, possibly saving lives and property. Thus, this information and the maps created with it could be utilized to regulate and prevent flooding. Furthermore, the findings of this study could be used to develop a value evaluation criterion for quantitative data used in the development of river master plans, land-use plans, and flood-protection plans. It's also necessary to conduct continual research on the many aspects of floods and the new models. Other machine learning and deep learning models, such as XGBoost, KNN, SVM, and the random forest model, may be used to show how the result varies through these models, and these models can be used to measure susceptibility in other flood-prone areas of the country. Several benefits can be gained by using machine learning models for regional-scale flood susceptibility mapping as opposed to more conventional approaches. Using machine learning models, such as ANN, to determine the flood-prone zone in a smaller region, such as Shylet, enabled a comparison of the changes to a larger scale like country. It also provides greater precision and variation than larger scales. Particularly useful for regional-level studies like Shylet where a wide variety of environmental and socio-economic factors are at play, this study produces a more accurate and detailed representation of flood susceptibility by incorporating complex relationships between variables and integrating multiple data sources. This study could be applied to Bangladesh's north-eastern region to control, manage, and lessen flash flood damage. The Haor region's flood preparedness program should be founded on thorough and educated planning. The quality and precision of the flood modeling that forms the basis of flood mitigation assessments, land use planning rules, physical infrastructure, and even flood insurance classifications all have a significant impact. It is anticipated that climate change will increase the frequency and severity of inundation events. Future research may contemplate incorporating future climate scenarios into the model in order to assess how flood susceptibility may change over time. Future research could be conducted to forecast flooding in forthcoming years and identify vulnerable regions. Finally, we are hopeful that the workflow outlined in this study will serve as a model for future growth in related fields.

## Author contribution statement

Rhyme Rubayet Rudra: Conceived and designed the experiments; Performed the experiments; Analyzed and interpreted the data; Contributed reagents, materials, analysis tools or data; Wrote the paper.

Showmitra Kumar Sarkar: Conceived and designed the experiments; Contributed reagents, materials, analysis tools or data; Wrote the paper.

## Data availability statement

Data will be made available on request.

## Declaration of competing interest

The authors declare that they have no known competing financial interests or personal relationships that could have appeared to influence the work reported in this paper.
